# P38 MAPK Signaling in the Retina: Effects of Aging and Age-Related Macular Degeneration

**DOI:** 10.3390/ijms241411586

**Published:** 2023-07-18

**Authors:** Natalia A. Muraleva, Nataliya G. Kolosova

**Affiliations:** Institute of Cytology and Genetics (ICG), Siberian Branch of Russian Academy of Sciences (SB RAS), 10 Akad. Lavrentieva Avenue, 630090 Novosibirsk, Russia; kolosova@bionet.nsc.ru

**Keywords:** aging, age-related macular degeneration, p38 MAPK, MAPK14, phosphorylation, OXYS rat

## Abstract

Age-related macular degeneration (AMD) is the leading cause of irreversible visual impairment worldwide. Age is the greatest risk factor for AMD but the underlying mechanism remains unascertained, resulting in a lack of effective therapies. Growing evidence shows that dysregulation of the p38 MAPK signaling pathway (SP) contributes to aging and neurodegenerative diseases; however, information about its alteration in the retina with age and during AMD development is limited. To assess the contribution of alterations in p38 MAPK signaling to AMD, we compared age-associated changes in p38 MAPK SP activity in the retina between Wistar rats (control) and OXYS rats, which develop AMD-like retinopathy spontaneously. We analyzed changes in the mRNA levels of genes of this SP in the retina (data of RNA-seq) and evaluated the phosphorylation/activation of key kinases using Western blotting at different stages of AMD-like pathology including the preclinical stage. p38 MAPK SP activity increased in the retinas of healthy Wistar rats with age. The manifestation and dramatic progression of AMD-like pathology in OXYS rats was accompanied by hyperphosphorylation of p38 MAPK and MK2 as key p38 MAPK SP kinases. Retinopathy progression co-occurred with the enhancement of p38 MAPK-dependent phosphorylation of CryaB at Ser59 in the retina.

## 1. Introduction

Age-related macular degeneration (AMD) is the neurodegenerative disease that is the third leading reason for blinding eye diseases worldwide [1]. Early stages of the disease are asymptomatic, which greatly complicates the diagnosis and treatment of AMD [2]. Hallmarks of AMD are the formation of extracellular drusen deposits (which are located between retinal pigment epithelium (RPE) cells and Bruch’s membrane), degeneration of retinal photoreceptors, and geographic atrophy of RPE, all of which can cause a loss of central visual acuity [3,4,5,6,7]. Experts predict that, by 2040, the number of patients with AMD will increase dramatically to 288 million people [8], while there is no effective treatment for this disease.

Oxidative stress, inflammation, and apoptosis play critical roles in the pathogenesis of AMD [9,10]. In recent years, many investigators have attempted to identify the intracellular signaling cascades that mediate these processes. Among other things, the mitogen-activated protein kinase (MAPK) pathway has proven to be a dominant player in many physiological phenomena, among them cell proliferation, differentiation, survival, and apoptosis [11,12].

MAPK signaling pathways (SPs) are categorized into four distinct groups: extracellular signal-regulated protein kinases 1 and 2 (ERK1 and -2), JNK1–3, p38, and ERK5 [13]. They are important mediators of signal transduction and are activated by diverse extracellular stimuli, including growth factors, mitogens, hormones, cytokines, and different cellular stressors such as oxidative stress, ultraviolet radiation, and hypoxia. MAPK SPs consist of three levels of kinases, the sequential phosphorylation of which leads to activation of the transcription factors located in the cytoplasm and nucleus, thereby initiating the expression of target genes and the formation of a biological response [14].

Alteration of proteostasis is considered one of the hallmarks of aging. Similar accumulation of pathological aggregates progresses during the development of age-related neurodegenerative diseases, which, in addition to AMD, include Alzheimer’s disease (AD). Furthermore, the accumulation of pathological aggregates of amyloid beta (Aβ) and of hyperphosphorylated tau protein, levels of neuroinflammation and oxidative stress, and other manifestations of AD have been shown to depend on the activity of MAPK kinases [15,16,17,18,19]. As a result, a type of AD treatment aimed at inhibiting the activity of the MAPK SP is being actively developed. The similarity of hallmarks between AD and AMD is suggestive of common mechanisms underlying the development of these diseases. It is possible that one of them is an alteration of MAPK SP activity. In agreement with this notion, despite the limited data about the impact of MAPKs’ activity on AMD development, pharmacological inhibition of these SPs is being discussed as a new avenue for the prevention and treatment of this disease and of other neurodegenerative conditions [12].

Recently, we showed that the AD-like pathology in OXYS rats develops and progresses dramatically against the background of alterations of ERK1/2 and p38 MAPK activities in the brain [20,21,22]. The first clinical manifestations of AMD-like retinopathy were detected in ~20% of OXYS rats during ophthalmoscopic examination at 5–6 weeks of age; at the age of 3–4 months, all the animals demonstrated symptoms of retinopathy. At this time point in OXYS rats, the first stage of the disease was prevalent. Four to five percent of the animals at this age had features of the second stage. The third stage was registered in 44% of these rats at age 12 months. By the age of 24 months, the changes were irreversible in nearly all animals. In another study, we found that the signs of AMD, which in OXYS rats develop spontaneously together with the signs of AD pathology, develop during an alteration of the activity of the ERK1/2 SP in the retina [23]. Nonetheless, how the activity of the p38 MAPK SP changes in the retina with aging and with the development of AMD-like pathology remains unknown.

In the present study, we attempted to decipher the role of p38 MAPK in age-related changes in the retina and in the development of AMD by analyzing the regulation of phosphorylation/enzyme activation in the p38 MAPK SP. Our results indicate that p38 MAPK activation is dynamically dysregulated with age. Moreover, the temporal correlation of MAPK activation with critical periods of manifestation and active progression of signs of AMD-like pathology in OXYS rats implies an important role of these proteins in the pathogenesis events of this disease.

## 2. Results

### 2.1. Differentially Expressed Genes (DEGs) Associated with the p38 MAPK SP in the Rat Retina

**Figure 1:**
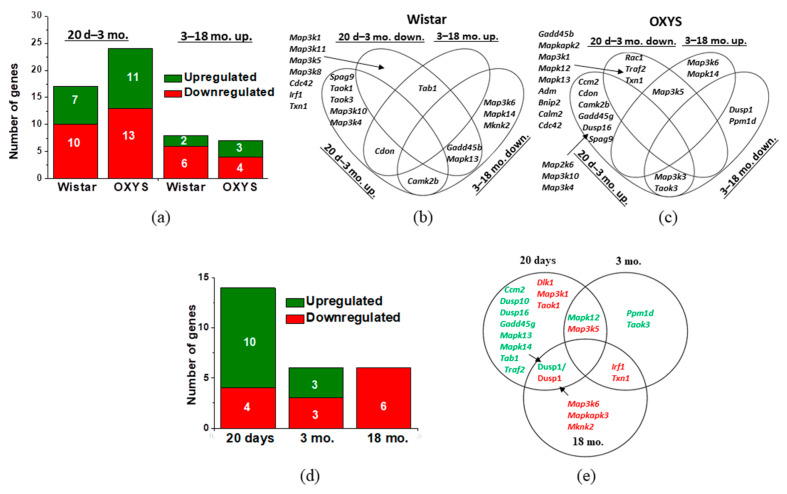
Age-related changes in the expression of genes of the p38 MAPK SP in the retina of Wistar and OXYS rats. Numbers of DEGs depending on age in Wistar and OXYS rats (**a**). DEGs of the p38 MAPK SP in Wistar (**b**) and OXYS (**c**) rats with age. Numbers of DEGs in 20-day-old and 3- and 18-month-old OXYS rats compared to age-matched Wistar rats (**d**). Differential expression means a comparison with the parental control strain (Wistar) (**e**). The data are highlighted in green for upregulation and in red for downregulation of genes.

First, we compared age-dependent changes in the expression of genes involved in the p38 MAPK SP in the retina in OXYS and Wistar rats. According to the analysis of the retinal transcriptome, among the 56 genes involved in the p38 MAPK SP (according to the Rat Genome Database), the expression of 17 genes changed in the Wistar retina from the age of 20 days to 3 months (Figure 1a, Supplementary Table S1). Among them, the mRNA expression of seven genes (*Camk2b*, *Cdon*, *Map3k10*, *Map3k4*, *Spag9*, *Taok1*, and *Taok3*) increased and that of ten genes (*Cdc42*, *Gadd45b*, *Irf1*, *Map3k1*, *Map3k11*, *Map3k5*, *Map3k8*, *Mapk13*, *Tab1*, and *Txn1*) decreased (Figure 1b). The expression of eight genes changed between ages 3 and 18 months. Among them, the mRNA expression of two genes (*Cdon* and *Tab1*) rose, while that of six genes (*Camk2b*, *Gadd45b*, *Map3k6*, *Mapk13*, *Mapk14*, and *Mknk2*) diminished (Figure 1b, Supplementary Table S1).

The largest number of DEGs (24) involved in the p38 MAPK SP of OXYS rats’ retinas was detected during the period of manifestation of signs of retinopathy similar to AMD (between ages of 20 days to 3 months). Among these DEGs, the expression of 11 genes (*Camk2b*, *Ccm2*, *Cdon*, *Dusp16*, *Gadd45g*, *Map2k6*, *Map3k10*, *Map3k3*, *Map3k4*, *Spag9*, and *Taok3*) increased and that of 13 (*Adm*, *Bnip2*, *Calm2*, *Cdc42*, *Gadd45b*, *Map3k1*, *Map3k5*, *Mapk12*, *Mapk13*, *Mapkapk2*, *Rac1*, *Traf2*, and *Txn1*) decreased (Figure 1a,c).

Active progression of the signs of AMD from 3 to 18 months of age occurred during expression changes of seven genes involved in the p38 MAPK SP. Among them, the mRNA levels of genes *Map3k5*, *Map3k6*, and *Mapk14* went up and the mRNA levels of four genes (*Dusp1*, *Map3k3*, *Ppm1d*, and *Taok3*) declined (Figure 1c, Supplementary Table S1).

A comparison of the age-associated dynamics of gene expression showed that, from the age of 20 days to 3 months, mRNA expression of 12 genes changed similarly between Wistar and OXYS rats. Among them, the mRNA expression of six genes (*Camk2b*, *Cdon*, *Map3k10*, *Map3k4*, *Spag9*, and *Taok3*) decreased and that of six genes (*Cdc42*, *Gadd45b*, *Map3k1*, *Map3k5*, *Mapk13*, and *Txn1*) increased. In addition, in Wistar rats, the expression of genes *Irf1*, *Map3k11*, *Map3k8*, and *Tab1* diminished, while the expression of *Taok1* rose.

According to the analysis of the retinal transcriptome from age 3 to 18 months, Wistar and OXYS rats did not share DEGs showing unidirectional alterations of expression. Nevertheless, in Wistar rats, mRNA expression of genes *Map3k6* and *Mapk14* went down, while in OXYS rats, it increased.

An analysis of interstrain differences revealed that, in the preclinical period (at the age of 20 days), 14 DEGs were detectable in OXYS rats compared to control (Wistar) rats (Figure 1d). The levels of mRNA of ten genes (*Ccm2*, *Dusp1*, *Dusp10*, *Dusp16*, *Gadd45g*, *Mapk12*, *Mapk13*, *Mapk14*, *Tab1*, and *Traf2*) went up and the levels of four genes (*Dlk1*, *Map3k1*, *Map3k5*, and *Taok1*) diminished (Figure 1e, Supplementary Table S2).

The manifestation of AMD-like pathology in 3-month-old OXYS rats occurred simultaneously with expression changes of six genes involved in the p38 MAPK SP: the mRNA expression of three genes (*Mapk12*, *Ppm1d*, and *Taok3*) increased and that of three genes (*Irf1*, *Map3k5*, and *Txn1*) declined (Figure 1e, Supplementary Table S2).

The dramatic progression of AMD-like pathology in 18-month-old OXYS rats was associated with six DEGs involved in the p38 MAPK SP (Supplementary Table S1). At the same time, the mRNA levels of genes *Dusp1*, *Irf1*, *Map3k6*, *Mapkapk3*, *Mknk2*, and *Txn1* were found to be reduced (Figure 1e, Supplementary Table S2).

### 2.2. Age-Related Changes in p38 MAPK and Phospho- (p-) p38 MAPK Amounts in the Rat Retina

**Figure 2:**
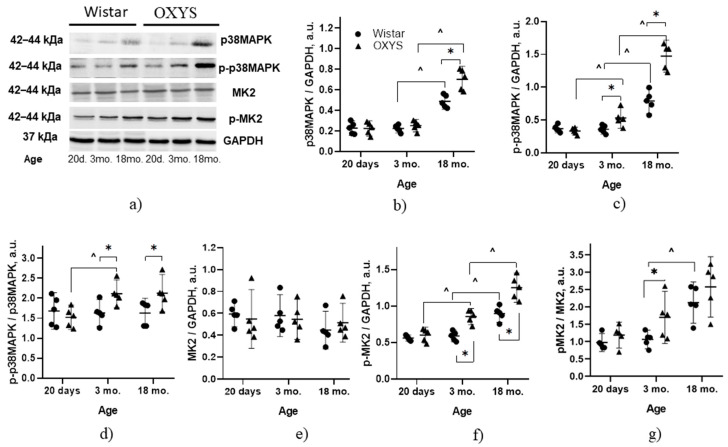
Protein amounts of p38 MAPK, p-p38 MAPK, MK2, and p-MK2 in the retinas of Wistar rats and OXYS rats at the ages of 20 days and 3 and 18 months. Representative Western blot images of proteins p38 MAPK and p-p38 MAPK (**a**). Graphical presentation of p38 MAPK (**b**) and p-p38 MAPK (**c**) protein levels and the ratio of p-p38 MAPK to p38 MAPK (**d**); MK2 (**e**) and p-MK2 (**f**) protein amounts and the ratio of p-MK2 to MK2 (**g**). The protein amounts were normalized to GAPDH. The results of six independent experiments are presented. The data are shown as a median with an interquartile range (q1–q3). ^ Significant differences from a previous age within a strain; * significant differences between the strains at the same age (*p* < 0.05).

Next, we compared p38 MAPK protein expression levels and the extent of p38 phosphorylation in the retina between Wistar and OXYS rats. According to an analysis of variance (ANOVA), the level of p38 MAPK in the retina depended on age (F_2,24_ = 123.2; *p* < 0.001, Figure 2a,b) and reached maximum values by the age of 18 months. On the other hand, the protein levels of p38 MAPK at ages 20 days and 3 months did not differ between Wistar and OXYS rats (*p* > 0.05). By the age of 18 months, the increase in the p38 MAPK amount proceeded at different rates, which led to a significant increase in this parameter in the retina of OXYS rats relative to Wistar rats of the same age (*p* < 0.004).

The level of p38 MAPK phosphorylation depended on the genotype (strain) of rats (F_1,24_ = 38.0; *p* < 0.001) and changed with age (F_2,24_ = 124.3; *p* < 0.001). Factors “genotype” and “age” interacted (F_1,24_ = 23.5; *p* < 0.001). A comparison of group means indicated that the level of p-p38 MAPK at the age of 20 days did not differ between the strains (*p* > 0.05); however, by the age of 3 months in OXYS rats, this level increased (*p* < 0.026) and became higher than that in Wistar rats (*p* < 0.008, Figure 2c). By the age of 18 months, the p-p38 MAPK content increased in rats of both strains but in OXYS rats, it remained higher than in Wistar rats (*p* < 0.049). Analysis of the p-p38 MAPK:p38 MAPK ratio uncovered a dependence on the genotype (F_1,24_ = 5.9; *p* < 0.023) and no change with age (F_2,24_ = 2.6; *p* = 0.097) in Wistar and OXYS rats. Factors “genotype“ and “age“ interacted (F_2,24_ = 3.7; *p* < 0.041). A comparison of group means suggested that the p-p38 MAPK:p38 MAPK ratio in the retina was higher in OXYS rats than in Wistar rats at the ages of 3 and 18 months (*p* < 0.025 and *p* < 0.049, respectively, Figure 2d).

### 2.3. Age-Related Changes in MK2 and p-MK2 Contents of the Rat Retina

Next, we compared the amounts of the MK2 protein (which is upstream of p38 MAPK in the SP) in Wistar and OXYS rats and the extent of its phosphorylation in the retina (Figure 2a,e,f). According to ANOVA, MK2 protein expression in the retinas of Wistar and OXYS rats does not depend on the genotype of the animals (F_1,24_ = 0.006; *p* = 0.94) and does not change with age (F_2,24_ = 1.07; *p* = 0.36).

The level of MK2 phosphorylation in the rat retina depended on the animals’ genotype (F_1,24_ = 38.7; *p* < 0.001) and changed with age (F_2,24_ = 68.6; *p* < 0.001). Factors “genotype” and “age” were found to interact (F_1,24_ = 7.0; *p* < 0.004). At the age of 20 days, the level of *p*-MK2 did not differ between the two strains (*p* > 0.05); however, by the age of 3 months, in OXYS rats, its level increased (*p* < 0.002) and became higher than that in Wistar rats (*p* < 0.001). By age 18 months, the ratio went up in rats of both strains but remained higher in OXYS rats than in Wistar rats (*p* < 0.003).

Analysis of the p-MK2:MK2 ratio revealed that it depends on the genotype (F_1,24_ = 7.5; *p* < 0.012) and age (F_2,24_ = 18.3; *p* < 0.001) but that the factors do not interact (F_1,24_ = 1.8; *p* = 0.188). A comparison of group means showed that the p-MK2:MK2 ratio at the age of 20 days did not differ between the two strains (*p* > 0.05). An increase in this ratio by the age of 3 months in OXYS rats (*p* < 0.037) led to a significant increase in this parameter relative to Wistar rats (Figure 2g). By the age of 18 months, the p-MK2:MK2 ratio rose in Wistar (*p* < 0.002) and OXYS (*p* < 0.020) rats and, in OXYS rats, this parameter did not exceed that in Wistar rats of the same age (*p* > 0.05).

### 2.4. Age-Related Changes in CryaB and p-CryaB Contents of the Rat Retina

**Figure 3:**
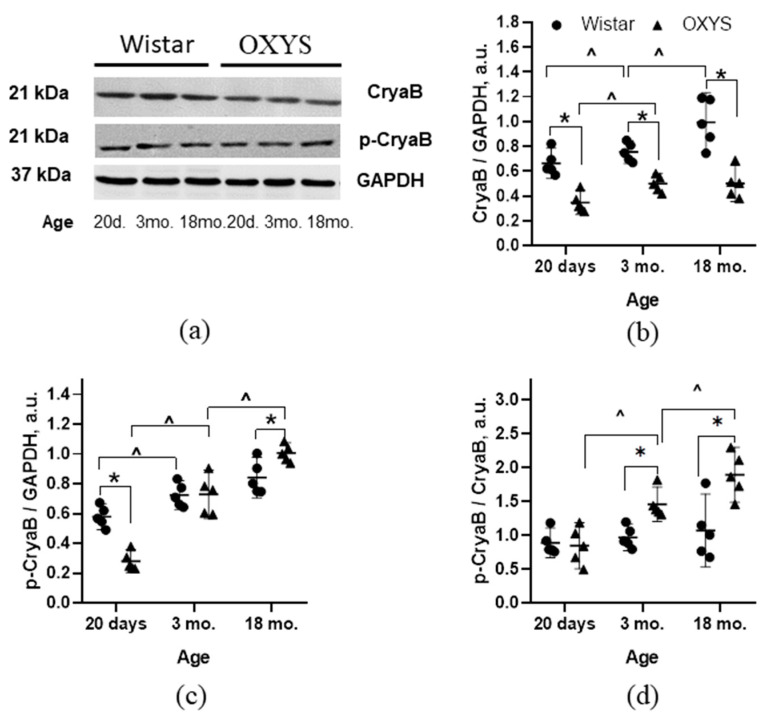
Protein amounts of CryaB and p-CryaB-S59 in the retinas of Wistar rats and OXYS rats at ages 20 days and 3 and 18 months. (**a**) Representative Western blot images of proteins. Graphical presentation of CryaB (**b**) and p-CryaB-S45 (**c**) protein levels, and of the ratio of p-CryaB-S59 to CryaB (**d**). The protein amounts were normalized to GAPDH. Results of six independent experiments are presented. The data are shown as a median (q1–q3). ^ Significant differences from a previous age within a strain; * significant differences between the strains at the same age (*p* < 0.05).

We examined the expression of molecular chaperone CryaB as a target protein phosphorylated by components of the p38 MAPK SP as well as the level of its phosphorylation on Ser59. According to the ANOVA, the concentration of CryaB in the retinas of Wistar and OXYS rats depends on the genotype of the animals (F_1,24_ = 73.4; *p* < 0.001) and changes with age (F_2,24_ = 11.4; *p* < 0.001). Factors “genotype” and “age” did not interact (F_1,24_ = 2.9; *p* = 0.07). In all age groups, the level of CryaB was lower in OXYS rats than in Wistar rats of the same age (*p* < 0.001, *p* < 0.001, and *p* < 0.001, respectively, Figure 3a,b).

The level of CryaB phosphorylation did not depend on the genotype of the animals (F_2,24_ = 1.8; *p* = 0.193) but changed with age (F_2,24_ = 78.3; *p* < 0.001). Factors “genotype” and “age” interacted (F_1,24_ = 17.5; *p* < 0.001). A comparison of group means indicated that the amount of p-CryaB at the age of 20 days was lower in OXYS rats than in Wistar rats (*p* < 0.001, Figure 3c). The level of p-CryaB in OXYS retinas reached the values seen in Wistar rats by the age of 3 months and became higher than that in Wistar rats (*p* < 0.018) at age 18 months.

Analysis of the p-CryaB/CryaB ratio showed that it depended on the genotype (F_1,24_ = 17.2; *p* < 0.001) and age (F_2,24_ = 12.2; *p* < 0.001) and that the factors interacted (F_1,24_ = 6.0; *p* < 0.007). A comparison of group means suggested that the p-CryaB/CryaB ratio at the age of 20 days did not differ between the two strains (*p* > 0.05). At the age of 3 months in OXYS rats, the p-CryaB/CryaB ratio exceeded that in Wistar rats insignificantly (*p* < 0.003) and led to a significant elevation of this parameter relative to Wistar rats (*p* < 0.010) at the age of 18 months (Figure 3d).

## 3. Discussion

Growing evidence shows that the biological processes that contribute to aging and age-related diseases are associated with dysregulation of MAPK SPs but information about the alterations of MAPKs in aging is still controversial [24]. In the current study, we assessed p38 MAPK signaling in the retinas of OXYS rats at different stages of AMD-like pathology, including the preclinical stage, with Wistar rats as a control. This approach allowed us to analyze for the first time changes of p38 MAPK SP activity in the retina with age and with the development of AMD-like pathology. We demonstrated that p38 MAPK SP activity increases during physiological aging in the retinas of Wistar rats. We also noticed that the manifestation and dramatic progression of AMD-like pathology in OXYS rats is accompanied by hyperphosphorylation of p38 MAPK and of MK2 as key p38 MAPK SP kinases.

In the first part of the work, according to transcriptomic data, we assessed changes in the mRNA levels of genes of this SP in the retinas of Wistar rats and OXYS rats. The transcriptome analysis did not reveal any significant changes in the activity of p38 MAPK SP with age in the retinas of either Wistar or OXYS rats at the mRNA levels. Alterations of the expression of genes involved in the p38 MAPK SP showed trends that were similar between the disease-free rats and OXYS rats having the AMD-like pathology. The number of downregulated genes exceeded the number of genes upregulated as compared to a previous age (Supplementary Table S1).

A comparison of transcriptome data from the retinas of Wistar and OXYS rats identified the p38 MAPK SP–related DEGs that may influence the development of the AMD-like pathology. Among them, mRNA expression of *Mapk12* (mitogen-activated protein kinase 12), *Mapk13* (mitogen-activated protein kinase 13) and *Mapk14* (mitogen-activated protein kinase 14) was increased in the retinas of 20-day-old OXYS rats before the manifestation of AMD-like pathology signs. These genes encode different isoforms of p38 MAPK. The upregulated *Gadd45g* (growth arrest and DNA damage-inducible gamma) and *Tab1* (TGF-beta-activated kinase 1/MAP3K7-binding protein 1) serve as p38 MAPK SP activators, mediating phosphorylation and subsequent activation of upstream kinases. The product of gene *TAB1* can activate MAPK14 and thus represents an alternative activation pathway in addition to the MAPKK pathways that promote MAPK14 biological responses to various stimuli. This result possibly indicates an increase in the activity of the p38 MAPK SP. Nevertheless, among the genes with elevated expression, we found *Dusp16* (dual specificity phosphatase 16), which performs negative regulation of the p38 MAPK SP [25]. Moreover, we also registered the underexpression of genes *Dusp1* (dual specificity phosphatase 1) and *Dusp10* (dual specificity phosphatase 10), which inactivate signal transduction, thus providing the desired balance via inhibition of p38 MAPK-mediated toxic effects and via maintenance of the protective mechanisms of MAPK signaling [25,26]. In addition, among the genes showing underexpression, there were genes that positively regulated the p38 MAPK SP: *Taok3* (TAO kinase 3) and *Map3k5* (mitogen-activated protein kinase kinase kinase 5). It is noteworthy that the mRNA level of the *Map3k1* gene (mitogen-activated protein kinase kinase kinase 1) is low in OXYS rats. It is an activator of the p38 MAPK SP with an antiapoptotic effect. Its downregulation is consistent with our previous data on the activation of apoptosis in the retinas of 20-day-old OXYS [27].

During the manifestation of AMD-like pathology signs, we found six DEGs taking part in the p38 MAPK SP. OXYS rats have a low level of *Map3k5* mRNA and a high level of *Ppm1d* (protein phosphatase Mg^2+^/Mn^2+^-dependent 1D) mRNA, the products of which inhibit p38 MAPK SP activity through dephosphorylation of upstream kinase RAF1 [28]. This finding possibly points to a decrease in the activity of this SP but we detected the upregulation of genes *Mapk12* and *Taok3*, which are activators of p38 MAPK. Among the analyzed DEGs that manifested downregulation here, *Irf1* (interferon-regulatory factor 1) is noteworthy. The protein it encodes participates in cell proliferation, apoptosis, and an immune response. Downregulation of the *Irf1* gene can alter an immune response in OXYS rats. It has been previously shown that the progression of the AMD-like pathology in OXYS rats is accompanied by reactive gliosis. On the other hand, the migration (typical for AMD) of activated macrophages and microglia into the photoreceptor layer in the period of either manifestation or progression of AMD-like retinopathy in OXYS rats is not observed, probably indicating a reduced immune response [29].

It is noteworthy that, during the dramatic progression of AMD-like pathology signs in OXYS rats, all the DEGs involved in the p38 MAPK SP turned out to be downregulated, though, among them, there were both activators (*Map3k6* and *Mapkapk3*) and inhibitors (*Dusp1*) of the p38 MAPK SP. Additionally, among them, most of the genes are downstream in the signaling cascade and do not affect the activity of this SP as a whole.

The activity of the p38 MAPK SP can be affected by mutations in the genes associated with this SP, for example, *BRAF* or *RAS*, which encode its upstream activators [30]. Recently, we found single-nucleotide polymorphisms in genes *Map3k7*, *Mapk14*, and *Spag9*, which are involved in the p38 MAPK SP in OXYS rats [31]. These polymorphisms are synonymous amino acid substitutions and do not significantly influence the structure or function of the transcript and/or protein or the activity of the p38 MAPK SP as a whole.

Signal transduction along the p38 MAPK SP is mediated by sequential phosphorylation of downstream kinases, which is an important characteristic of SP activity. Accordingly, it is the increase in the level of phosphorylated p38 MAPK that indicates the activation of this signaling. We compared the transcriptome data with protein amounts of key kinases and their phosphorylated forms. Our results suggest that the levels of p38 MAPK and p-p38 MAPK change with aging in the retina in control (Wistar) rats. Their levels increased by 18 months of age. Our study complements the study on p38 MAPK activity in the rat retina by Oliveira and coauthors [32], where only rats at ages between P0 (postnatal day zero) and P45 were used. Those authors demonstrated that the p38 MAPK SP plays a significant role in the postnatal development of the visual system in rats. The peak of p38 MAPK phosphorylation was registered at P4, which corresponds to the process of programmed cell death, which determines the physiological development of the retina. The second peak occurred during the opening of the eyes (P15) and the completion of retinal development. It is likely that hyperphosphorylation of p38 MAPK in the Wistar retina with age may be related to the accumulation of pathological aggregates and to the associated cellular stress that develops with physiological aging.

Although MARK SPs were recently recognized as potential targets for the treatment of AMD [4], possible changes in p38 MARK phosphorylation in the retina during the development of AMD have been unknown before our study. We found only one study on the amount of this protein in the retina in AMD: data from Dridi et al. revealed no differences in the levels and phosphorylation of p38 MAPK in the retinas of patients in the late stages of AMD as compared with non-AMD eyes [33]. In our study, the p38 MAPK and p-p38 MAPK protein amounts rose in the retinas of senescence-accelerated OXYS rats; these changes occurred at a young age and coincided with the period of manifestation and dramatic progression of the AMD-like pathology.

Furthermore, as a criterion for assessing p38 MAPK signaling activity, we evaluated alterations of phosphorylation of downstream target protein CryaB. This small protein chaperone is crucial for the response of retinal cells to oxidative stress, which is an important factor in the pathogenesis of AMD [34,35]. It is known that p38 MAPK regulates CryaB phosphorylation at the Ser59 position by stimulating MK2 (MAPK-activated protein kinase 2). CryaB is one of the important substrates of p38 MAPK and is independently regulated by the p38 MAPK SP, followed by downstream phosphorylation events and the regulation of the cellular functions of these proteins [36,37].

Notably, CryaB and MK2 hyperphosphorylation coincided with an increase in p38 MAPK phosphorylation in the retinas of Wistar and OXYS rats, thereby confirming the activation of the p38 MAPK SP. The phosphorylation of CryaB enhances its ability to form strong bonds with neurotoxic proteins, including Aβ, and renders CryaB insoluble. Similar results have been obtained in brain homogenates of OXYS rats [20,21] and patients with AD [38], where this phenomenon was associated with the response to the accumulation of pathologically aggregated proteins. Thus, our data indicate that the activity of the p38 MAPK SP increases in the retina with age in both healthy Wistar rats and in senescence-accelerated OXYS rats. On the other hand, in OXYS rats, not only progression but also manifestation of the AMD-like pathology occurs simultaneously with hyperactivation of the p38 MAPK SP in comparison with Wistar rats. These results are consistent with our previous finding of the accumulation of pathological amyloid aggregates and tau hyperphosphorylation in the retinas of OXYS rats [23]. It is worth mentioning that, in OXYS rats, which in parallel spontaneously develop AMD-like and AD-like pathologies, p38 MAPK hyperactivation in the retina coincides in time with p38 MAPK hyperphosphorylation in the brain, thereby possibly indicating a commonality of the mechanisms underlying these two diseases.

## 4. Materials and Methods

### 4.1. Ethics Statement

All animal procedures were approved by Scientific Council No. 9 of the ICG SB RAS according to Directive 2010/63/EU of the European Parliament and Council as of 22 September 2010.

### 4.2. Animals

Male senescence-accelerated OXYS rats (*n* = 39) and age-matched male Wistar rats (control, *n* = 39) at ages of 20 days and 3 and 18 months were obtained from the Center for Genetic Resources of Laboratory Animals at the ICG SB RAS (RFMEFI61914X0005 and RFMEFI61914X0010). At age 4 weeks, the pups were weaned, housed in groups of five animals per cage (57 × 36 × 20 cm^3^), and kept under standard laboratory conditions (22 ± 2 °C, on a 12 h light/12 h dark cycle, with lights on at 9 a.m.). The animals had access to standard rodent feed (PK-120-1; Laboratorsnab, Ltd., Moscow, Russia) and water ad libitum.

### 4.3. Western Blotting

Retinas from OXYS and Wistar rats at ages of 20 days and 3 and 18 months (*n* = 6) were lysed with RIPA Lysis Buffer containing protease inhibitors (Sigma-Aldrich, St. Louis, MO, USA) and phosphatase inhibitors. The protein concentration was determined by the BCA assay (Thermo Fisher, Waltham, MA, USA). Proteins in these extracts were separated by electrophoresis and transferred onto a nitrocellulose membrane (Bio-Rad, Heracles, CA, USA), which was next blocked with 1% BSA for 1 h and then incubated overnight at 4 °C with a relevant primary antibody: an anti-MK2 (phospho T334) antibody, anti-Alpha B crystallin antibody, anti-CryaB phospho S59 antibody, anti-MK2 antibody (E341), and anti-GAPDH (Abcam, Eugene, OR, USA; cat. # ab63378, ab76467, ab5577, ab32567, and ab8245, respectively) and anti-phospho-p38 MAPK alpha and anti-p38 MAPK alpha antibodies (cat. # 36-8500 and 33-1300, Invitrogen, Carlsbad, CA, USA). After an immunoreaction with a corresponding secondary antibody (1:5000; cat. # ab6721 and ab97046; Abcam, Boston, MA, USA), bands of proteins were detected by means of a ChemiDoc MP Imaging System (Bio-Rad, Hercules, CA, USA). GAPDH and β-actin were employed to set up a loading internal control.

### 4.4. Gene Expression Analysis

The retinas of OXYS and Wistar rats at 20 days and 3 and 18 months of age (*n* = 3 animals per strain (i.e., genotype) and age) were used for RNA-Seq analysis. The sequencing data were preprocessed using the Cutadapt tool (https://cutadapt.readthedocs.io, http://www.genoanalytica.ru/, accessed on 1 September 2021) to remove adapters and low-quality sequences. The resulting reads were mapped onto the Rnor_6.0 reference genome assembly in the TopHat2 software (https://ccb.jhu.edu/software/tophat/, accessed on 1 September 2021). The data were then converted into gene count tables by means of ENSEMBL and RefSeq gene annotations data. The resulting tables were subjected to the analysis of differential gene expression in the DESeq software (https://bioconductor.org/packages/release/bioc/html/DESeq2.html, accessed on 1 September 2021). The genes with *p* < 0.05 were selected as differentially expressed. The analysis included genes associated with the p38 MAPK SP according to the Rat Genome Database (https://rgd.mcw.edu/, accessed on 20 September 2021) and the Kyoto Encyclopedia of Genes and Genomes (http://www.genome.jp/kegg/, accessed on 20 September 2021).

### 4.5. Statistical Analysis

The data were processed using ANOVA (STATISTICA 10.0, Statsoft, Tulsa, OK, USA). An ANOVA with the subsequent post hoc Newman–Keuls test was carried out when ANOVA assumptions were satisfied; otherwise (that is, with a non-normal distribution of the data in the studied groups), a nonparametric Kruskal–Wallis ANOVA was conducted with subsequent multiple comparisons of mean ranks for all the groups. Independent variables for two-way ANOVA were either treatment or age and genotype. The data are shown as mean ± SE. Data were assumed to be significant at *p* < 0.05.

## Data Availability

All data necessary to reproduce the results are contained within the article. Raw data are available upon request.

## Supplementary Materials

The following supporting information can be downloaded at https://www.mdpi.com/article/10.3390/ijms241411586/s1.
