# Supplementary material for: P38 MAPK Signaling in the Retina: Effects of Aging and Age-Related Macular Degeneration
Source: Int J Mol Sci. 2023 Jul 18;24(14):11586. doi: 10.3390/ijms241411586 (PMC10380409; doi:10.3390/ijms241411586)
Supplement: Supplementary file 1 [file ijms-24-11586-s001.zip › ijms-2493184-supplementary.pdf]

Supplementary Table S1. DEGs of the p38 MAPK SP in the retina of Wistar and OXYS rats depending on age of the rats.

Gene symbol	Gene name	log2	p value	p_{adj}
	**Wistar 20 d. - Wistar 3 mo.**			
Camk2b	*calcium/calmodulin-dependent protein kinase II beta*	0.334	1.20×10^{-05}	0.001
Cdon	*cell adhesion associated, oncogene regulated*	0.620	4.81×10^{-08}	1.12×10^{-06}
Map3k10	*mitogen activated protein kinase kinase kinase 10*	0.577	1.07×10^{-08}	2.96×10^{-07}
Map3k4	*mitogen activated protein kinase kinase kinase 4*	0,218	0.004	0.017
Spag9	*sperm associated antigen 9*	0.267	8.91×10^{-06}	0.0001
Taok1	*TAO kinase 1*	0.300	0.001	0.002
Taok3	*TAO kinase 3*	0.631	4.95×10^{-06}	6.49×10^{-05}
Cdc42	*cell division cycle 42*	-0.454	0.0001	0.001
Gadd45b	*growth arrest and DNA-damage-inducible, beta*	-0.600	2.96×10^{-05}	0.0003
Irf1	*interferon regulatory factor 1*	-1.281	5.56×10^{-09}	1.66×10^{-07}
Map3k1	*mitogen-activated protein kinase kinase kinase 1*	-0.609	2.40×10^{-08}	6.03×10^{-07}
Map3k11	*mitogen-activated protein kinase kinase kinase 11*	-1.087	0.001	0.004
Map3k5	*mitogen-activated protein kinase kinase kinase 5*	-0.397	0.005	0.020
Map3k8	*mitogen-activated protein kinase kinase kinase 8*	-0.990	0.003	0.012
Mapk13	*mitogen activated protein kinase 13*	-1.308	8.56×10^{-11}	3.96×10^{-09}
Tab1	*TGF-beta activated kinase 1/MAP3K7 binding protein 1*	-0.226	0.004	0.017
Txn1	*thioredoxin 1*	-1.613	1.37×10^{-15}	1.58×10^{-13}
	**Wistar 3 mo. - Wistar 18 mo.**			
Cdon	*cell adhesion associated, oncogene regulated*	0.305	0.016	0.278
Tab1	*TGF-beta activated kinase 1/MAP3K7 binding protein 1*	0.201	0.023	0.335
Camk2b	*calcium/calmodulin-dependent protein kinase II beta*	-0.208	0.008	0.193
Gadd45b	*growth arrest and DNA-damage-inducible, beta*	-0.386	0.009	0.211
Map3k6	*mitogen-activated protein kinase kinase kinase 6*	-0.631	0.001	0.036
Mapk13	*mitogen activated protein kinase 13*	-0.596	0.003	0.117
Mapk14	*mitogen activated protein kinase 14*	-0.192	0.008	0.192
Mknk2	*MAP kinase-interacting serine/threonine kinase 2*	-0.384	0.037	0.418
	**OXYS 20 d - OXYS 3 mo.**			
Camk2b	*calcium/calmodulin-dependent protein kinase II beta*	0.678	4.03×10^{-06}	4.42×10^{-05}
Ccm2	*CCM2 scaffold protein*	0.425	0.001	0.004
Cdon	*cell adhesion associated, oncogene regulated*	0.584	4.65×10^{-07}	6.51×10^{-06}
Dusp16	*dual specificity phosphatase 16*	0.234	0.032	0.089
Gadd45g	*growth arrest and DNA-damage-inducible, gamma*	0.517	0.004	0.015
Map2k6	*mitogen-activated protein kinase kinase 6*	0.300	0.034	0.093
Map3k10	*mitogen activated protein kinase kinase kinase 10*	0.510	3.76×10^{-07}	5.37×10^{-06}
Map3k3	*mitogen activated protein kinase kinase kinase 3*	0.296	0.001	0.002
Map3k4	*mitogen activated protein kinase kinase kinase 4*	0.330	1.47×10^{-05}	0.001
Spag9	*sperm associated antigen 9*	0.284	2.51×10^{-06}	2.90×10^{-05}
Taok3	*TAO kinase 3*	0.386	0.005	0.020
Adm	*adrenomedullin*	0.417	1.98×10^{-08}	3.84×10^{-07}
Bnip2	*BCL2 interacting protein 2*	-0.166	0.006	0.022
Calm2	*calmodulin 2*	-0.207	0.001	0.004
Cdc42	*cell division cycle 42*	-0.640	7.13×10^{-08}	1.21×10^{-06}
Gadd45b	*growth arrest and DNA-damage-inducible, beta*	-0.666	3.72×10^{-06}	4.11×10^{-05}
Map3k1	*mitogen activated protein kinase kinase kinase 1*	-0.731	5.08×10^{-11}	1.69×10^{-09}

Map3k5	*mitogen activated protein kinase kinase kinase 5*	-0.322	0.029	0.082
Mapk12	*mitogen activated protein kinase 12*	-0.350	0.008	0.028
Mapk13	*mitogen activated protein kinase 13*	-0.840	2.59×10^{-05}	0.001
Mapkapk2	*mitogen-activated protein kinase-activated protein kinase 2*	-0.393	0.018	0.055
Rac1	*Rac family small GTPase 1*	-0.619	0.037	0.099
Traf2	*Tnf receptor-associated factor 2*	-0.223	0.045	0.116
Txn1	*thioredoxin 1*	-0.867	0.008	0.029
**OXYS 3 mo. - OXYS 18 mo.**				
Map3k5	*mitogen activated protein kinase kinase kinase 5*	-0.450	0.003	0.176
Map3k6	*mitogen activated protein kinase kinase kinase 6*	-0.422	0.027	0.420
Mapk14	*mitogen activated protein kinase 14*	-0.167	0.020	0.376
Dusp1	*dual specificity phosphatase 1*	0.430	0.023	0.394
Map3k3	*mitogen activated protein kinase kinase kinase 3*	0.203	0.025	0.414
Ppm1d	*protein phosphatase, Mg^{2+}/Mn^{2+}-dependent, 1D*	0.202	0.023	0.401
Taok3	*TAO kinase 3*	0.339	0.016	0.344

Supplementary Table S2. DEGs of the p38 MAPK SP in the retina of 20-day-old and 3- and 18-month-old OXYS rats compared to age-matched Wistar rats.

Gene symbol	Gene name	Age	Wistar	OXYS	log2	p value	p_{adj}
Ccm2	*CCM2 scaffold protein*	20 d.	388.6	461.8	0.243	0.037	0.208
Dusp1	*dual specificity phosphatase 1*	20 d.	396.2	517.2	0.360	0.048	0.241
Dusp10	*dual specificity phosphatase 10*	20 d.	134.8	165.0	0.286	0.008	0.079
Dusp16	*dual specificity phosphatase 16*	20 d.	275.0	359.0	0.377	0.001	0.003
Gadd45g	*growth arrest and DNA-damage-inducible. gamma*	20 d.	256.5	353.3	0.436	0.010	0.090
Mapk12	*mitogen-activated protein kinase 12*	20 d.	476.4	607.0	0.3382	0.008	0.078
Mapk13	*mitogen-activated protein kinase 13*	20 d.	80.6	128.5	0.617	0.002	0.026
Mapk14	*mitogen-activated protein kinase 14*	20 d.	955.3	1111.8	0.217	0.001	0.010
Tab1	*TGF-beta activated kinase 1/MAP3K7 binding protein 1*	20 d.	440.4	495.5	0.169	0.014	0.113
Traf2	*Tnf receptor-associated factor 2*	20 d.	238.9	277.2	0.211	0.039	0.212
Dlk1	*delta like non-canonical Notch ligand 1*	20 d.	184.9	51.49	-1.098	0.003	0.037
Map3k1	*mitogen-activated protein kinase kinase kinase 1*	20 d.	364.6	312.4	-0.218	0.039	0.212
Map3k5	*mitogen-activated protein kinase kinase kinase 5*	20 d.	209.0	151.4	-0.449	0.001	0.015
Taok1	*TAO kinase 1*	20 d.	2946.4	2453.2	-0.261	0.001	0.017
Mapk12	*mitogen-activated protein kinase 12*	3 mo.	598.8	779.6	0.368	0.006	0.114
Ppm1d	*protein phosphatase Mg^{2+}/Mn^{2+}-dependent 1D*	3 mo.	526.5	617.9	0.227	0.011	0.157
Taok3	*TAO kinase 3*	3 mo.	1000.7	1230.4	0.287	0.041	0.327
Irf1	*interferon regulatory factor 1*	3 mo.	482.7	239.1	-0.914	4.58×10^{-5}	0.003
Map3k5	*mitogen-activated protein kinase kinase kinase 5*	3 mo.	278.5	190.7	-0.524	0.001	0.024
Txn1	*thioredoxin 1*	3 mo.	2948.7	1191.8	-0.980	0.003	0.067
Dusp1	*dual specificity phosphatase 1*	18 mo.	553.2	402.9	-0.426	0.024	0.293
Irf1	*interferon regulatory factor 1*	18 mo.	440.8	258.3	-0.100	0.002	0.068
Map3k6	*mitogen-activated protein kinase kinase kinase 6*	18 mo.	162.0	122.6	-0.422	0.034	0.341
Mapkapk3	*mitogen-activated protein kinase-activated*	18 mo.	294.0	198.6	-0.214	0.044	0.384

	protein kinase 3						
Mknk2	*MAP kinase-interacting serine/threonine kinase 2*	18 mo.	1295.3	866.2	-0.131	0.003	0.095
Txn1	*thioredoxin 1*	18 mo.	1592.9	1191.8	-0.307	0.016	0.235
